# Mechanisms of Anti‐Inflammatory and Antioxidant Activity of Extracts of Selected South African Celastraceae and Crassulaceae Plant Species With Known Efficacy Against Bovine Mastitis Bacterial Pathogens

**DOI:** 10.1155/sci5/2796708

**Published:** 2026-02-23

**Authors:** E. C. Ogbuadike, S. M. Nkadimeng, E. T. Khunoana, C. C. Igwe, D. N. Qekwana, I. M. Petzer, L. J. McGaw

**Affiliations:** ^1^ Department of Paraclinical Sciences, Faculty of Veterinary Sciences, University of Pretoria, Private Bag X04 Onderstepoort, Pretoria, 0110, South Africa, up.ac.za; ^2^ Federal Institute of Industrial Research, Oshodi (FIIRO), P.M.B, Ikeja, 21023, Lagos, Nigeria, fiiro.org; ^3^ Department of Production Animal Studies, Faculty of Veterinary Sciences, University of Pretoria, Private Bag X04 Onderstepoort, Pretoria, 0110, South Africa, up.ac.za

**Keywords:** anti-inflammatory, antioxidant, *Bryophyllum pinnatum*, *Kalanchoe gunniae*

## Abstract

Inflammation, a complicated reaction to microbial infection or injury, promotes healing under normal circumstances. However, when it becomes uncontrolled, it can result in cell damage or even death. Inflammation is a major feature of infectious mastitis in cattle as well as in humans, with substantial health, welfare and financial challenges. Uncontrolled inflammation is commonly accompanied by excess reactive oxygen species (ROS), which may be harmful. Ethanol and acetone leaf extracts of *Bryophyllum pinnatum* (Lam.) Oken (synonym: *Kalanchoe pinnata* (Lam.) Pers.), *Kalanchoe gunniae* Gideon F. Sm. and Figueiredo*, Maytenus undata* (Thunb.) Blakelock and *Maurocenia frangula* Mill. were investigated for their antioxidant and anti‐inflammatory activities. In previous work, these plant species had promising antimicrobial activity against mastitis‐causing bacteria. In this study, anti‐inflammatory activity of the extracts was investigated in terms of their ability to inhibit the action of the enzymes implicated in inflammation, namely 15‐lipoxygenase (15‐LOX) and cyclooxygenase (COX). Their ability to inhibit the production of nitric oxide (NO) by lipopolysaccharide (LPS)‐activated RAW 264.7 macrophages was also tested. Using ELISA kits, the effect of the plant extracts on the regulation of cytokine production was determined. Evaluation of antioxidant activity was done using electron reducing 2, 2′‐azino‐bis (3‐ethylbenzothiazoline‐6‐sulfonic acid) (ABTS) and radical scavenging 2, 2‐diphenyl‐1‐picrylhydrazyl (DPPH) assays. The best 15‐LOX inhibitory activity with IC_50_ values of 1.25 and 2.03 μg/mL for acetone and ethanol extracts, respectively, was observed with *K. gunniae* extracts. The best NO inhibition of 80.48% and cell viability of 96.75% was produced by the acetone extract of *B. pinnatum* at the highest concentration (100 μg/mL). Significant inhibition of COX‐2 was observed with both extracts of *K. gunniae* and *B. pinnatum*. In this study, the highest inhibitory activity against proinflammatory cytokines and enhanced production of anti‐inflammatory cytokines were observed with extracts of *B. pinnatum*. *Kalanchoe gunniae* extracts had the best antioxidant activity with IC_50_ values ranging from 0.06 to 0.42 μg/mL. This research highlights the therapeutic potential of the selected plant species in managing inflammation and oxidative stress associated with bacterial mastitis.

## 1. Introduction

Bovine mastitis, commonly caused by bacterial pathogens, is a multifactorial disease characterised by inflammation of the mammary glands, resulting in decreased milk production and quality [1]. It is a major pathological condition in dairy herds, with reduced production and heightened culling rates contributing to significant economic losses [2]. In bovines, following chronic inflammation when the immune system is not able to eliminate the pathogen, the infection does not clear and the inflammatory response persists, leading to visible mastitis symptoms, i.e., clinical mastitis [3]. Clinical cases of mastitis are characterised by visible clinical signs in the milk, udder or system of the animal, while subclinical cases are detected by a somatic cell count (SCC) exceeding 200,000 cells/mL in milk, as well as the presence of microbial organisms in milk [4, 5]. The majority of mastitis cases are caused by bacteria, with *Staphylococcus aureus* being one of the predominant species [6–8].

Acute inflammation associated with mastitis is necessary to control infection as it assists in removing bacteria and cellular debris. However, dysregulation of inflammatory processes can lead to prolonged or chronic inflammation, which contributes to cell and tissue damage rather than healing [9]. Excess activation of phagocytes during inflammation results in increased oxygen consumption by the neutrophils [10]. This then leads to oxidative stress burst (oxidative burst) at the site of microbial invasion [11]. Following this, reactive oxygen species (ROS) are generated including free radicals such as superoxide (O_2_^.^), hydroxyl (^.^OH) and peroxyl (^.^OOH, ROO^.^), as well as the nonradical species hydrogen peroxide (H_2_O_2_). [12]. Excess production of these free and nonfree radicals harms the surrounding tissue either by powerful direct oxidising action with O_2_ or indirectly with H_2_O_2_ and OH radicals formed from O_2_^.^[13]. This complex process also results in an increase in several inflammation‐related enzymes, including phospholipase A_2_ (PLA_2_), lipoxygenases (15‐LOX and 5‐LOX), cyclooxygenases (e.g. COX‐1 and COX‐2) and inducible nitric oxide synthase (iNOS) [14]. Increased iNOS then enzymatically oxidises L‐arginine to citrulline, forming nitric oxide (^−^NO). The O_2_ reacts with ^−^NO to form peroxynitrite (ONOO^−^), which is a strong oxidant able to initiate lipid peroxidation [14]. An inflammatory response is triggered, leading to the production of more ROS‐generating enzymes and activation of the transcription factor, nuclear factor kappa B (NFkB), proinflammatory cytokines and other inflammatory mediators, such as tumour necrosis factor (TNF‐α) [15]. More membrane destruction and tissue damage takes place, leading to increased and persistent inflammation [11].

A previous study reported that *Maytenus undata* (Thunb.) Blakelock and *Maurocenia frangula* Mill. from the Celastraceae family, and *Kalanchoe gunniae* Gideon F. Sm. and Figueiredo and *Bryophyllum pinnatum* (Lam.) Oken (Crassulaceae) exhibited good antibacterial activities against various *Staphylococcus aureus* strains [16]. These test strains included reference ATCC strains and isolates from clinical cases of mastitis in dairy cows. Building on those findings, this study focuses on evaluating the *in vitro* antioxidant and anti‐inflammatory properties of these plant extracts. The aim is to explore their potential anti‐inflammatory mechanisms, which may be relevant for managing inflammation associated with diseases such as bovine mastitis. In the present study, *in vitro* antioxidant and anti‐inflammatory efficacy of the selected plant extracts was tested using various bioassays. The effect of the plant extracts on NO release from macrophages, inflammatory enzymes (15‐LOX and COX) and selected pro‐ and anti‐inflammatory cytokines were assayed.

## 2. Materials and Methods

### 2.1. Collection of Plants, Drying and Extraction

*Maytenus undata* was collected from the Walter Sisulu Botanical Garden (South African National Biodiversity Institute, SANBI), Gauteng, South Africa. *Maurocenia frangula* was collected from the Lowveld National Botanical Garden (SANBI), Nelspruit, Mpumalanga, South Africa. *Kalanchoe gunniae* was collected from the Pretoria Botanical Garden (SANBI), South Africa. *Bryophyllum pinnatum* was collected from the Federal Institute of Industrial Research, Oshodi, Lagos Nigeria, Medicinal Plant Garden. The herbarium specimen of *Maytenus undata* was deposited at the H.G.W.J. (Herold Georg Wilhelm Johannes) Schweickerdt Herbarium, University of Pretoria. Herbarium samples of *Bryophyllum pinnatum* and *Kalanchoe gunniae* were deposited at the SANBI Herbarium, Pretoria Botanical Garden, South Africa, and their voucher specimen numbers were also obtained. The voucher numbers are shown in Table 1.

**TABLE 1:** Voucher numbers of plant species investigated.

Plant family	Plant species	Voucher number
Celastraceae	*Maytenus undata* (Thunb.) Blakelock	PRU 125486
	*Maurocenia frangula* Mill.	PRE 1004262

Crassulaceae	*Kalanchoe gunniae* Gideon F. Sm. and Figueiredo	PRE 1004266
	*Kalanchoe pinnata* (Lam.) Pers. Synonym: *Bryophyllum pinnatum* (Lam.) Oken	PRE 1004263

Collected leaves of *M. undata* and *M. frangula* were dried indoors at room temperature (25°C ± 2°C) in open mesh loosely woven bags. *Bryophyllum pinnatum* and *Kalanchoe gunniae* leaves were cut into pieces as they were more fleshy, and dried in a dehumidifying oven drier at 33°C. The dried, ground leaves were extracted with acetone (≥ 99% from Minema Chemicals (Pty) Ltd, South Africa) and ethanol (99.9% from Minema Chemicals (Pty) Ltd, South Africa) using a modified ultrasonication method based on Sserunkuma et al. [17]. Briefly, quantities of 4 g of ground plant material were extracted separately with 40 mL of acetone and 40 mL of ethanol by shaking the samples vigorously for 25 min with a mechanical shaker and then sonicating in an ultrasonicator for 15 min. It was decided to use both acetone and ethanol as extraction solvents, firstly because acetone extracts a broad range of polar and nonpolar compounds, and has been recommended for preparing plant extracts for antibacterial testing [18]. Secondly, ethanol extracts more polar compounds from plant material and is commonly preferred for use in preparing samples for formulation and product development as it is less flammable than acetone [19]. Each of the extracts was filtered through Whatman No 1 filter paper into a glass vial. The plant material residues were re‐extracted with 20 mL of the extracting solvent, and the filtrates from the three extractions were combined. The extracts were dried by evaporating the solvents under a stream of air at 30°C.

### 2.2. Antioxidant Activity

#### 2.2.1. 1,1‐Diphenyl‐2‐Picrylhydrazyl (DPPH) Assay

The antioxidant assay was performed using the DPPH radical scavenging assay according to the method described by Re et al. [20] with some modifications [21]. Briefly, 40 μL of methanol (Minema) was added to each well of a U‐bottom 96‐well microtitre plate (Lasec). Thereafter, 40 μL of the extracts (1 mg/mL in methanol) was added to 4 wells in the second row of the microtitre plate and serially diluted from 100 to 1.56 μg/mL. Freshly prepared DPPH with an optical density of 0.91–0.99 at 517 nm was added (160 μL) into the two wells of each sample, while 160 μL of methanol was added into the other two wells to be used as a blank. The plate was allowed to stand at room temperature for 30 min in the dark, and absorbance was measured at 517 nm using a microplate reader (Biotek, Synergy HT). The positive controls used were Trolox, which is a water‐soluble analogue of vitamin E, and ascorbic acid (vitamin C). The experiments were repeated three times with three replicates in each experiment. The percentage of inhibition of DPPH oxidation was calculated according to the following formula:

(1) % DPPH inhibition=Absorbance control−Absorbance sampleAbsorbance control×100.

The antioxidant ability was expressed in IC_50_ values as the concentration of the sample necessary to scavenge DPPH by 50%.

For plant extracts, it has been established that IC_50_ < 10 μg/mL is regarded as strong radical scavenging activity, IC_50_ from 10 to 50 μg/mL is good antioxidant activity, IC_50_ > 50 and ≤ 100 μg/mL represents moderate activity, and IC_50_ > 100 and ≤ 250 μg/mL is regarded as weak activity [22–24].

#### 2.2.2. 2,2′‐Azinobis‐(3‐Ethylbenzothiazoline‐6‐Sulphonic Acid) (ABTS) Assay

The ABTS scavenging activity of the extracts was assessed using the method of Brand‐Williams et al. [25] with modifications. Ascorbic acid and Trolox were used as positive controls. Briefly, the ABTS solution was prepared with potassium persulphate (Sigma‐Aldrich) in equal amounts and allowed to react for 16 h at room temperature in the dark before use. Then, 40 μL of methanol was added to all the wells of a U‐bottom 96‐well microtitre plate. Subsequently, 40 μL of extracts (1 mg/mL in methanol) was added to each of 4 wells in the second row of the microtitre plate and serially diluted in the same manner as above with DPPH. Freshly prepared ABTS with an optical density of 0.71–0.79 at 734 nm was added (160 μL) into two wells of each sample, while methanol was added into the other two wells used as blanks. The ABTS scavenging ability of the extracts was measured in terms of a colour change after 5‐min incubation in the dark. The experiments were repeated three times, and IC_50_ values were calculated according to the formula:

(2) % ABTS inhibition=Absorbance control−Absorbance sampleAbsorbance control×100.

Antioxidant activity was expressed in IC_50_ values as the concentration of the sample necessary to scavenge ABTS by 50%.

### 2.3. Anti‐Inflammatory Activity

#### 2.3.1. Inhibition of 15‐LOX

The anti‐inflammatory activity of extracts was evaluated *in vitro* using the soybean 15‐LOX inhibitory assay [26, 27]. Briefly, the extracts were diluted to 10 mg/mL in dimethyl sulphoxide (DMSO) and then reconstituted to 2 mg/mL in buffer (Tris‐HCL buffer, 50 mM, pH 7.4). Quercetin was used as a positive control, also prepared in DMSO to a concentration of 10 mg/mL and diluted in buffer to 1 mg/mL. To all the wells, 20 μL of the buffer was added. Then, to the first row was added 20 μL of the extracts, followed by serial dilution, resulting in test concentrations of 100–0.78 μg/mL. The enzyme, 15‐LOX (Sigma‐Aldrich), was prepared in buffer to a concentration of 0.1 mg/mL and 40 μL of this solution was added to each well before incubation for 5 min. Approximately 40 μL of the substrate, linoleic acid (140 μM) (Sigma‐Aldrich), was added to all the wells except for the blank and incubated for 20 min. Afterwards, 100 μL of freshly prepared FOX reagent (prepared with xylenol orange and ferrous sulphate) was added and incubated for 30 min. Then, 40 μL of the substrate was added into the blank wells just before reading the absorbance at 560 nm. The experiments were done in triplicate and repeated twice. The percentage of inhibition was calculated using the following formula:

(3) % enzyme inhibition=Absorbance sample−absorbance blankAbsorbance negative control−absorbance blank×100.

IC_50_ values were determined using linear regression from the graph plotted using % enzyme inhibition against the concentrations of the extracts and quercetin.

#### 2.3.2. Inhibition of NO Production

RAW 264.7 macrophage cells purchased from the American Type Culture Collection (ATCC TIB‐71, USA) were used. The RAW cells were cultured in Dulbecco’s modified Eagle’s medium (DMEM) (Pan Biotech, Separations) supplemented with 1% streptomycin (100 μg/mL) (Celtic Molecular Diagnostics) and 1% penicillin (100 units/mL) and 10% foetal bovine serum (Gibco, Sigma) at 37°C in an atmosphere of 5% CO_2_ (HeraCell 150, Thermo Electron Corp., USA). Seeding of the RAW cells was done at a density of 4 × 10^4^ cells/well into each well of columns 2–11 of sterile tissue culture‐treated 96‐well plates (NEST, Whitehead Scientific). The plates containing the seeded macrophages were incubated at 37°C in 5% CO_2_ for 24 h. Then, the medium was aspirated from all the wells and replaced with fresh medium. Different concentrations of the extracts (1.6–100 μg/mL) were added to the wells, except for the control wells and the blank wells. Then, 1 μg/mL of lipopolysaccharide (LPS) (Sigma‐Aldrich) was aliquoted into each well to treat the cells. The control wells contained only media and LPS, whereas the blank wells contained only the media. All the plates were then incubated again for 24 h.

Griess reagent (Sigma) was used to measure the amount of nitrite produced in the culture media [28]. Nitrite, being a stable metabolite of NO, was used to indicate the production of NO. After 24‐h incubation, 100 μL of cultured media from each of the wells was transferred into new replica plates. Then, 100 μL of Griess reagent (Sigma‐Aldrich) was added to each of the wells and the plates were incubated at room temperature in the dark for 15 min. Using a microplate reader (Biotek, Synergy HT), absorbance was measured at 540 nm. In every experiment, blanks were included in wells of columns 1 and 12, which contained only media and Griess reagent. A sodium nitrite (NaNO_2_) standard curve was used to calculate the amount of nitrite in the culture media.

Cytotoxicity on the remaining RAW 264.7 cells following removal of the supernatant for use in the NO assay was measured using the tetrazolium‐based colorimetric (methyl tetrazolium bromide [MTT]) assay. Viability of cells was determined using the MTT assay described by Mosmann [29] with modifications. After 24‐h incubation of all the well plates (extract treated/controls/blanks) with 1 μg/mL LPS, the media was aspirated from all the wells. Prewarmed phosphate‐buffered saline (PBS, 200 μL) was used to rinse each well, followed by adding 100 μL of fresh medium to each well. Then, 40 μL of MTT (from a stock solution of 5 mg/mL dissolved in PBS) was added and the plates were incubated 37 C for 4 h in 5% CO_2_. Medium from all the cells was aspirated. To solubilise the formazan salt precipitate, 50 μL of DMSO was added and the plates were shaken for at least 1 min on a plate shaker. Using a microplate reader, absorbance was measured at a wavelength of 540 nm. A reference absorbance at a wavelength of 630 nm was also measured. Treated cells were compared to untreated cells by calculating the percentage of dead cells from the treated cells relative to the untreated cells. To calculate the percentage viability of cells, the following formula was used:

(4) % viability=Absorbance sampleAbsorbance control×100.

Each sample was tested in quadruplicate, and the experiments were repeated two times.

#### 2.3.3. Inhibition of COX‐2 and Cytokines

The effects of the extracts on COX‐2 were measured using the method of Noreen et al. [30] modified by du Toit et al. [31] and Nkadimeng et al. [21]. Briefly, when confluent, the RAW 264.7 macrophages were plated into several 25‐cm^2^ tissue culture flasks (NEST, Whitehead Scientific) at a density of 1 × 10^6^ cells per flask and incubated for 24 h. The medium was removed, fresh medium was added, and the cells were stimulated with LPS (1 μg/mL) and treated with different concentrations of the extracts. Quercetin, a well‐known antioxidant and a flavonol found in many fruits and plants, and N‐nitro‐L‐arginine methyl ester (L‐NAME), a NOS inhibitor, were used as positive controls. Control cells were stimulated with LPS but not treated, while the normal cells were cells which were neither stimulated with LPS nor treated with extracts. After 24 h, the medium was removed and stored in a −80°C freezer until the time of analysis.

##### 2.3.3.1. COX‐2 Enzyme Measurements

The effects of the extracts on COX‐2 were determined using the mouse PTGS2/COX‐2 prostaglandin endoperoxide synthase 2 (PGE_2_) ELISA kit (Elabscience, Biocom Africa) according to the manufacturer’s protocol. Concentrations of mouse COX‐2 in the cell culture media samples were calculated from the standard curve. The absorbance was directly proportional to the concentration of PGE_2_ in the sample medium.

##### 2.3.3.2. Cytokine Measurements

The effects of the extracts on levels of proinflammatory cytokines, TNF‐α and interleukin‐1 beta (IL‐1β) and anti‐inflammatory cytokine interleukin‐10 (IL‐10) were determined and quantified using the mouse ELISA kits with catalogue numbers E‐EL‐M0049, E‐EL‐M0037 and E‐EL‐M0046 (Elabscience, Biocom Africa), respectively, following the manufacturer’s instructions.

### 2.4. Statistical Analysis

Processing and analysis of obtained data were done with analysis of variance (ANOVA) in Microsoft Excel, SigmaStat and SPSS. Pairwise multiple comparison was done using the Holm–Sidak method. Results were stated as mean ± standard deviations. Statistically significant values were compared, and *p* value of ≤ 0.05 was considered statistically significant.

## 3. Results

### 3.1. Antioxidant Activity

Of the eight extracts prepared from four plants, *Kalanchoe gunniae* extracts had the best scavenging activity for both ABTS and DPPH assays (Table 2). *Kalanchoe gunniae* extracts had potent scavenging activity with IC_50_ below 1 μg/mL for both ABTS and DPPH assays. *Kalanchoe gunniae* ethanol extract was the most active for ABTS (IC_50_ = 0.06 ± 0.007 μg/mL) and DPPH (IC_50_ = 0.13 ± 0.007 μg/mL). Similar to the ethanol extract, the acetone extract of *Kalanchoe gunniae* also had very strong activity in the ABTS (IC_50_ = 0.30 ± 0.01 μg/mL) and DPPH assays (IC_50_ = 0.42 ± 0.034 μg/mL) (Table 2). The ethanol extracts of *Bryophyllum pinnatum, M. frangula* and *M. undata* had strong activity in both ABTS and DPPH assays. However, there was no significant difference (*p* ≤ 0.05) between the IC_50_ values of the ethanol extracts of *B. pinnatum, M. frangula, M. undata* and *Kalanchoe gunniae* extracts compared to the positive controls.

**TABLE 2:** Antioxidant assays of selected plant extracts with DPPH and ABTS.

Plant extracts/samples	Plant abbreviation	Extractant	DPPH—IC_50_ (μg/mL)	ABTS—IC_50_ (μg/mL)
*Maytenus undata*	*M*. *undata*	ethanol	**9.43 ± 0.17**	**4.41 ± 0.23**
		acetone	118.50 ± 11.81^∗^	47.51 ± 6.99^∗^

*Maurocenia frangula*	*M. frangula*	ethanol	**4.92 ± 0.77**	**6.79 ± 1.21**
		acetone	**9.37 ± 0.43**	16.68 ± 0.65

*Kalanchoe gunniae*	*K. gunniae*	ethanol	**0.13 ± 0.007**	**0.06 ± 0.007**
		acetone	**0.42 ± 0.034**	**0.30 ± 0.01**

*Bryophyllum pinnatum*	*B*. *pinnatum*	ethanol	**6.78 ± 0.55**	**7.04 ± 0.38**
		acetone	38.14 ± 5.97^∗^	47.38 ± 8.16^∗^

Trolox			**0.58 ± 0.02**	**0.46 ± 0.03**

Ascorbic acid			**0.19 ± 0.004**	**0.22 ± 0.01**

*Note:* DPPH = 2,2 diphenyl‐1‐picrylhydrazyl and ABTS = 2,2′‐azino‐bis (3‐ethylbenzothiazoline‐6‐sulfonic acid). Bolded = strong activity, IC_50_ below 10 μg/mL.

^∗^Statistically significant difference (*p* ≤ 0.05) when compared with controls (Trolox and ascorbic acid).

Regarding the acetone extracts of *B. pinnatum, M. frangula* and *M. undata*, *M. frangula* acetone extract had strong activity in the DPPH assay (9.37 ± 0.43 μg/mL) and good activity in the ABTS assay (16.68 ± 0.65 μg/mL). There was no statistically significant difference between the activities of the *M. frangula* acetone extract when compared to the activities of the positive controls. *M*. *undata* acetone extract and *B. pinnatum* acetone extracts had good to moderate activity with IC_50_, ranging from 47.51 ± 6.99 to 118.50 ± 11.81 μg/mL for both ABTS and DPPH, and these acetone extracts of these plants had significant differences when compared to the positive controls.

### 3.2. Anti‐Inflammatory Activity

#### 3.2.1. Inhibition of 15‐LOX

The 15‐LOX inhibitory activities of the plant extracts are presented in Table 3. The IC_50_ of the plant extracts ranged from 1.25 ± 0.02 μg/mL (strong activity) to 119.18 ± 0.99 μg/mL (weak activity). The lower the IC_50_, the higher the anti‐inflammatory activity. As with antioxidant activity, *K. gunniae* extracts also had the best 15‐LOX inhibitory activity with IC_50_ values of 1.25 and 2.03 μg/mL for the acetone and ethanol extracts, respectively. Extracts of *K. gunniae* were more potent than the positive control quercetin (IC_50_ = 4.93 μg/mL), but there was no statistically significant difference (*p* ≤ 0.05) between them.

**TABLE 3:** Anti‐inflammatory activity of selected plant extracts against lipoxygenase (LOX).

Plant	Extractant	LOX—IC_50_ (in μg/mL)
*Kalanchoe gunniae*	**Ethanol**	**2.03 ± 0.13**
	**Acetone**	**1.25 ± 0.02**

*Bryophyllum pinnatum*	Ethanol	18.99 ± 0.57
	Acetone	48.51 ± 5.12^∗^

*Maurocenia frangula*	Ethanol	17.26 ± 0.42
	Acetone	27.50 ± 0.47

*Maytenus undata*	Ethanol	13.26 ± 0.29
	Acetone	119.18 ± 0.99^∗^

Quercetin		**4.93 ± 0.28**

*Note:* LOX = 15‐lipoxygenase. Bold = strong activity, IC_50_ below 10 μg/mL.

^∗^Statistically significant difference (*p* ≤ 0.05) when compared with the control (quercetin).

The ethanol extracts of the other three plants (*B. pinnatum, M. frangula* and *M. undata*) had good inhibitory activity with IC_50_ values ranging from 13.26 to 18.99 μg/mL. The acetone extracts had good to weak enzyme inhibitory activity ranging from 27.50 to 119.18 μg/mL. With IC_50_ values of 48.51 and 119.18 μg/mL for acetone extracts of *B. pinnatum* and *M. undata*, respectively, these extracts were statistically significantly active (*p* ≤ 0.05) when compared with the positive control quercetin.

#### 3.2.2. Inhibition of NO Production and Effect on Macrophage Viability

**FIGURE 1:**
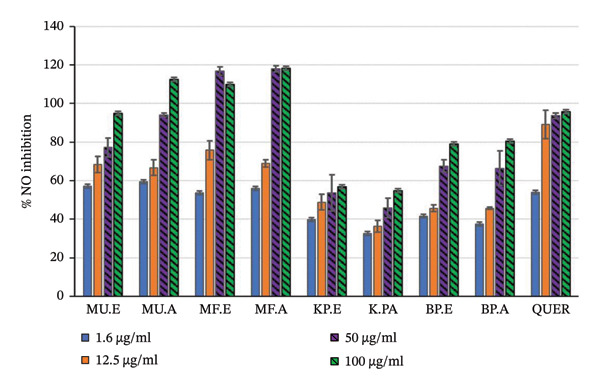
Anti‐inflammatory assay of selected plant extracts to determine the % inhibition of nitric oxide (NO) MU. E = *M. undata* ethanol extract, MU. A = *M. undata* acetone extract, MF. E = *M*. *frangula* ethanol extract, MF. A = *M. frangula* acetone extract, KP. E = *K. gunniae* ethanol extract, KP. A = *K. gunniae* acetone extract, BP. E = *B. pinnatum* ethanol extract, BP. A = *B. pinnatum* acetone extract, quer = quercetin.

**FIGURE 2:**
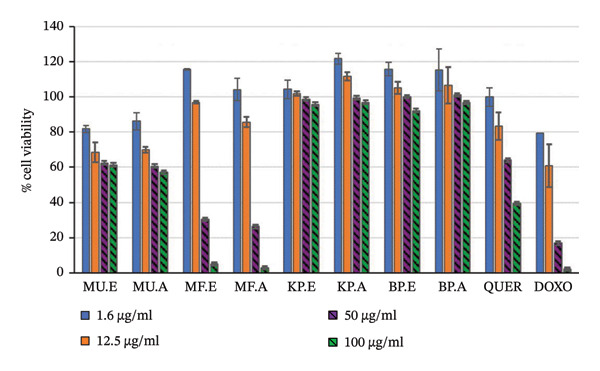
Effect of the selected plant extracts on the cell viability of treated raw macrophage cells, following nitric oxide inhibition MU. E = *M. undata* ethanol extract, MU. A = *M. undata* acetone extract, MF. E = *M*. *frangula* ethanol extract, MF. A = *M. frangula* acetone extract, KP. E = *K. gunniae* ethanol extract, KP. A = *K. gunniae* acetone extract, BP. E = *B. pinnatum* ethanol extract, BP. A = *B. pinnatum* acetone extract, quer = quercetin, doxo = doxorubicin.

In this study, the effects of plant extracts on NO production in LPS‐activated RAW 264.7 macrophages and the viability of cells following treatment were determined. The LPS induced the production of NO in the activated RAW 264.7 macrophages. Measurement of nitrite, which is a stable oxidising product of NO, correlates with the amount of NO produced. The inhibition of NO by the plant extracts and the effect of the plant extracts on cell viability of LPS‐activated RAW 264.7 macrophages showed concentration‐dependent effects (Table 4, Figures 1 and 2).

**TABLE 4:** Anti‐inflammatory activity of selected plant extracts against nitric oxide (NO) release and viability of treated RAW 264.7 macrophages.

Extracts	Conc. μg/mL	IC_50_ (μg/mL)	% NO inhibition	% macrophage cell viability
*Maurocenia frangula* ethanol	1.6	1.43 ± 0.20	53.77 ± 1.68	115.52 ± 0.37
	12.5		75.72 ± 4.88	96.82 ± 0.69
	50		116.88 ± 2.09	30.44 ± 1.18
	100		109.81 ± 13.07	5.10 ± 0.007

*Maurocenia frangula* acetone	1.6	1.62 ± 0.08	55.89 ± 8.47	104.14 ± 6.45
	12.5		69.03 ± 1.88	85.63 ± 2.80
	50		118.0 ± 1.68	26.35 ± 1.32
	100		118.17 ± 1.08	2.88 ± 0.004

*Maytenus undata* ethanol	1.6	0.91 ± 0.15	57.19 ± 4.45	81.71 ± 2.14
	12.5		68.35 ± 4.26	68.56 ± 5.60
	50		77.28 ± 4.91	62.45 ± 5.24
	100		94.96 ± 4.39	61.35 ± 0.18

*Maytenus undata* acetone	1.6	1.25 ± 0.14	59.55 ± 3.30	82.58 ± 4.99
	12.5		66.66 ± 4.05	68.66 ± 1.65
	50		94.08 ± 1.07	60.89 ± 0.30
	100		112.55 ± 0.11	57.62 ± 0.76

*Kalanchoe gunniae* ethanol	1.6	49.62 ± 7.86	39.95 ± 0.43	104.20 ± 5.14
	12.5		48.90 ± 4.06	101.78 ± 1.34
	**50**		**53.64 ± 9.47**	**98.65 ± 1.40**
	**100**		**56.73 ± 7.03**	**95.75 ± 3.50**

*Kalanchoe gunniae* acetone	1.6	76.25 ± 7.37	32.50 ± 4.04	121.58 ± 3.22
	12.5		36.49 ± 3.04	111.75 ± 2.40
	50		45.90 ± 5.00	99.38 ± 8.39
	**100**		**54.71 ± 5.14**	**96.88 ± 3.99**

*Bryophyllum pinnatum* ethanol	1.6	19.23 ± 2.64	41.69 ± 4.40	115.64 ± 3.78
	12.5		45.66 ± 1.74	105.16 ± 3.53
	**50**		**67.58 ± 3.24**	**99.99 ± 5.68**
	**100**		**78.93 ± 0.32**	**92.19 ± 0.41**

*Bryophyllum pinnatum* acetone	1.6	23.51 ± 3.97	37.57 ± 0.30	115.16 ± 11.86
	12.5		45.70 ± 0.47	106.41 ± 10.29
	**50**		**66.48 ± 8.99**	**101.15 ± 9.35**
	**100**		**80.48 ± 1.87**	**96.75 ± 3.94**

Quercetin	1.6	NA	39.54 ± 4.31	95.68 ± 2.89
	12.5		63.97 ± 3.77	93.70 ± 1.48
	50		83.35 ± 7.66	89.18 ± 7.32
	100		99.82 ± 5.42	53.84 ± 6.37

Doxorubicin	2	NA	NA	79.36 ± 9.34
	4		NA	60.76 ± 12.09
	10		NA	16.92 ± 0.88
	20		NA	2.14 ± 0.28

*Note:* Bold = extracts that gave NO inhibition above 50% with cell viability above 90%.

Abbreviation: NA, not applicable.

At the highest concentration tested (100 μg/mL), extracts of *B. pinnatum* had the best NO inhibition activity, ranging from 78.93% to 80.48% and the % cell viability ranged from 92.19% to 96.75% on the LPS‐activated macrophages. *Kalanchoe gunniae* extracts were the second best for NO inhibition activity. At the highest concentration tested, *K. gunniae* extracts had good inhibition activity ranging from 54.71% to 56.73% with cell viability ranging from 95.75% to 96.88%.

For *M. undata* extracts, at the highest concentration, *M. undata* ethanol extract showed NO inhibition activity of 94.96% and low cell viability of 61.35%. Also, at the highest concentration, *M. undata* acetone extract showed activity above 100% with low cell viability of 57.62%. Similar to the activities of *M. undata* extracts, *M. frangula* extracts showed activity above 100% with low cell viability. The cell viability observed with *M. frangula* extracts at the highest concentration tested was extremely low and ranged from 2.88% to 5.10%, similar to the effect of the negative control doxorubicin, which showed cell viability of 2.14% at the highest concentration tested.

#### 3.2.3. Inhibition of COX‐2 Enzyme

**FIGURE 3:**
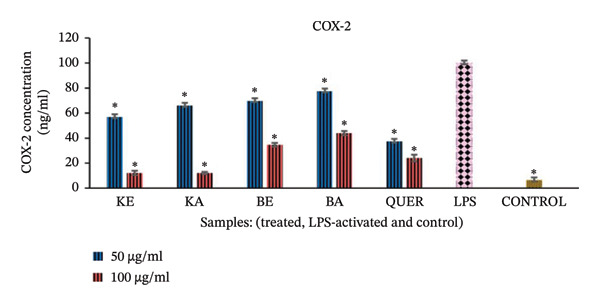
Effect of the selected plant extracts on LPS‐induced COX‐2 concentration for a period of 24 h KE = *K. gunniae* ethanol extract, KA = *K. gunniae* acetone extract, BE = *B. pinnatum* ethanol extract, BA = *B. pinnatum* acetone extract, quer = quercetin (positive control), LPS = differentiated and LPS‐induced COX‐2, control = differentiated cells that were neither induced with LPS nor treated, ∗ = statistically significant.

The results of the COX‐2 inhibition in the LPS‐activated RAW 264.7 macrophages are presented in Figure 3. Compared to the control normal cells (inactivated and untreated cells), the LPS‐activated macrophages were significantly induced to increase the concentration of COX‐2 (*p* < 0.001). At both concentrations used for the treatment, all the plant extracts showed very good activity and significantly inhibited the production of COX‐2 (*p* < 0.001) compared to the LPS‐activated cells. The plant extracts had similar effects as the positive control, quercetin (*p* < 0.001). Extracts of *K. gunniae* (100 μg/mL) were the most active, with more than 80% inhibition of COX‐2 production, more than that achieved with the positive control, quercetin.

#### 3.2.4. Effect of Extracts on Cytokine Production

##### 3.2.4.1. Inhibitory Effects of the Extracts on Proinflammatory Cytokine TNF‐α

**FIGURE 4:**
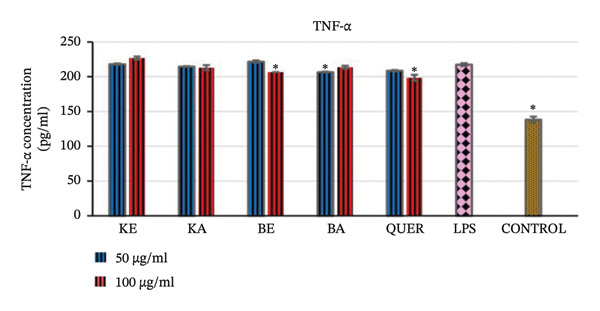
Effect of the selected plant extracts on LPS‐induced TNF‐α, proinflammatory cytokine production in RAW macrophages for a period of 24 h KE = *K. gunniae* ethanol extract, KA = *K. gunniae* acetone extract, BE = *B. pinnatum* ethanol extract, BA = *B. pinnatum* acetone extract, quer = quercetin (positive control), LPS = differentiated and LPS‐induced COX‐2, control = differentiated cells that were neither induced with LPS nor treated, ∗ = statistically significant.

The LPS‐activated RAW 264.7 macrophages significantly increased production of TNF‐α compared to the control normal cells (*p* < 0.001). The positive control quercetin (100 μg/mL) reversed this effect significantly (*p* < 0.001). *B. pinnatum* ethanol extract (100 μg/mL) and *B. pinnatum* acetone extract (50 μg/mL) also reversed the effect of LPS inhibiting the production of TNF‐α significantly (*p* < 0.038) and (*p* < 0.036), respectively. Extracts of *K. gunniae* were not able to significantly reverse the activated production of TNF‐α. The results of the LPS activation and the effect of the plant extracts are presented in Figure 4.

##### 3.2.4.2. The Effects of the Extracts on Proinflammatory Cytokine IL‐1β

**FIGURE 5:**
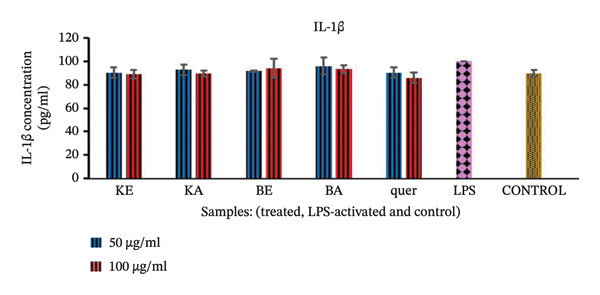
Effect of the selected plant extracts on LPS‐induced IL‐1β proinflammatory cytokine production in RAW macrophages for a period of 24 h KE = *K. gunniae* ethanol extract, KA = *K. gunniae* acetone extract, BE = *B. pinnatum* ethanol extract, BA = *B. pinnatum* acetone extract, quer = quercetin (positive control), LPS = differentiated and LPS‐induced COX‐2, control = differentiated cells that were neither induced with LPS nor treated, ∗ = statistically significant.

Compared to the control normal cells, the LPS‐activated macrophages increased the secretion of IL‐1β (Figure 5), but none of the extracts could significantly decrease secretion of IL‐1β.

##### 3.2.4.3. The Effects of the Extracts on Anti‐Inflammatory Cytokine IL‐10

**FIGURE 6:**
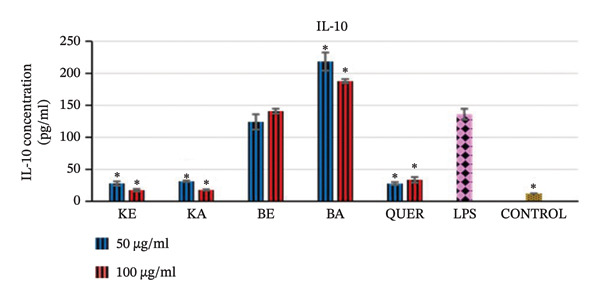
Effect of the selected plant extracts on LPS‐induced IL‐10 anti‐inflammatory cytokine concentration in RAW macrophages for a period of 24 h KE = *K. gunniae* ethanol extract, KA = *K. gunniae* acetone extract, BE = *B. pinnatum* ethanol extract, BA = *B. pinnatum* acetone extract, quer = quercetin (positive control), LPS = differentiated and LPS‐induced COX‐2, control = differentiated cells that were neither induced with LPS nor treated, ∗ = statistically significant.

In comparison to the control normal cells, the LPS‐activated RAW 264.7 macrophages significantly increased the production of IL‐10 (*p* > 0.001) as demonstrated in Figure 6. *Bryophyllum pinnatum* acetone extract (100 and 50 μg/mL) had the best activity and increased the production of IL‐10 significantly (*p* < 0.001) in comparison to the LPS‐activated cells. These were followed by *B. pinnatum* ethanol extract (100 and 50 μg/mL), which maintained the production of IL‐10 almost at the level of LPS‐activated cells. Extracts of *K. gunniae* and the positive control quercetin significantly (*p* < 0.001) decreased the production of IL‐10 compared to the LPS‐activated cells; however, they were still higher than the control normal cell levels.

## 4. Discussion

### 4.1. Antioxidant Activity

As inflammation is a major feature of mastitis disease pathology [17], substances that have radical scavenging properties can be very useful in controlling and inhibiting pathological inflammation, thereby aiding healing in inflammation‐related diseases [21]. In this study, plant extracts were tested to determine their ability to scavenge the free radicals, DPPH and ABTS. Lower concentrations of extracts that can cause 50% inhibition/reduction of the radicals mean higher scavenging activity of that extract [32]. *Kalanchoe gunniae* extracts (both ethanol and acetone) had the best scavenging activity, while for the other plants, ethanol extracts had superior activity to the acetone extracts. This indicates that the ethanol and acetone extracts of each of these plants most likely contain very different phytochemical profiles and this is worthy of future exploration. The antioxidant activity of the active extracts implies that they contain phytochemicals that exert their activity through the reactive oxygen subpathway of the arachidonic acid pathway and are capable of stopping the generation of free radicals (ROS), nonfree radicals and products of lipid oxidation, which are mediators of uncontrolled inflammation [27, 33].

Bhatti et al. [34] reported IC_50_ values of 94 and 120 μg/mL for ethanol and acetone extracts of *B. pinnatum* using the DPPH antioxidant assay method. However, in the current study, *B. pinnatum* ethanol and acetone extracts had strong activity. The different ranges of activity observed in their study could be a result of their use of the Soxhlet extraction method [34]. Due to high temperatures applied in Soxhlet extraction, the method is not suitable for any thermolabile phytochemical present in the plant material [35]. Thus, bioactive compounds/molecules present in the plant material may break down, decompose, disintegrate or change into nonactive or less active forms. In this study, extraction was done with ultrasonication at room temperature (25°C). Similar to the observation in this study that for antioxidant assays, ethanol extracts were generally more active than acetone extracts, Abubakar and Haque [35] also reported that ethanol extracts were more active compared to acetone extracts.

Upon investigating the effect of solvent polarity on the phenolic and antioxidant capacity of extracts of *Isatis tinctoria* (woad) of the family Brassicaceae, Wakeel et al. [36] concluded that the polarity of the solvent used has a significant influence on the extraction, polyphenol content and antioxidant activities. The results showed that solvent polarity greatly affects the yield of total phenolics and total flavonoids, which mainly increased with increasing solvent polarity index and suddenly decreased at very high polarity [36]. The total phenolic content was directly correlated with reducing power, antioxidant activity and free radical scavenging capacity [36].

Ahmed et al. [37] reported IC_50_ values of 3.48 ± 0.07 and 7.89 ± 0.31 μg/mL for DPPH and ABTS assays, respectively, for the crude extract of *M. undata* extracted with a mixture of acidified 70% acetone and n‐hexane. With the presence of water in the extractant mixture they used, their extractant was more polar than pure acetone. They reported strong activities in both the DPPH and ABTS assays, similar to the potent activities obtained in this study with the ethanol (polar) extract of *M. undata* in both DPPH and ABTS assays.

Reports on the antioxidant activity of *K. gunniae* are lacking. This is most likely because the plant is still not widely known as it was only recorded for the first time as a nothospecies in South Africa in 2019 [38], and extensive bioactivity research on the plant is yet to take place. Also, no available reports were found relating to the antioxidant activity of *M. frangula* extracts.

### 4.2. Anti‐Inflammatory Activity

Through the LOX inflammatory pathway, the 15‐LOX enzyme mediates the synthesis of the inflammation mediator, leukotriene, from arachidonic acid [39]. The LOX assay was conducted to determine the ability of the extracts to inhibit LOX enzyme activity. Extracts that are able to inhibit LOX activity are anticipated to be good anti‐inflammatory agents [40].

Extracts of *K. gunniae* had excellent 15‐LOX inhibitory activity with IC_50_ values comparable to that of the positive control. The ethanol extracts of the other plants (*B. pinnatum, M. frangula* and *M. undata*) had good anti‐15‐LOX activity, with much lower activity demonstrated by the acetone extracts. This affirms that the ethanol and acetone extracts of each of these plants most likely contain very different plant constituents. The observed 15‐LOX inhibition of the active extracts indicates that these extracts may act against inflammatory mediators in the arachidonic acid inflammatory pathway, which controls the secretion of 15‐LOX, or directly against 15‐LOX enzyme secretion in the 15‐LOX pathway, which is a subpathway of the arachidonic acid pathway [27].

Previous work by Ahmed et al. [37] reported moderate 15‐LOX inhibition of the acidified 70% acetone extract of *M. undata*, which confirms that different solvents are able to extract different phytochemicals with varying activity. Reports are lacking on the anti‐inflammatory activity of *M. frangula* and *K. gunniae*. Thus, to the best of our knowledge, the work in this study is the first report of the anti‐inflammatory activity of these two plant species. Reports are deficient on *in vitro* anti‐inflammatory activity of *B. pinnatum*, but Ojewole [41] reported significant *in vivo* activity of the aqueous extract of *B. pinnatum,* which inhibited fresh egg albumin‐induced acute inflammation in rats.

#### 4.2.1. Anti‐Inflammatory Assay Through Inhibition of NO Production and Effect on Cell Viability

Following the stimulation of macrophages with inflammatory mediators like LPS, iNOS is expressed [42]. Increased iNOS leads to enzymatic oxidation of l‐arginine to l‐citrulline, thereby forming NO. ROS like O_2_ react with NO to form ONOO^−^, which, as a strong oxidant, is able to initiate lipid peroxidation [14]. An inflammatory response is triggered, leading to the production of more ROS‐generating enzymes [9, 9] and proinflammatory cytokines like TNF‐α [15]. Thus, NO production in the cells leads to more oxidation or production of proinflammatory cytokines or a combination of both.

Substances which can inhibit NO production and yet are not toxic to macrophages are considered good anti‐inflammatory agents [40]. For plant extracts, effects on RAW 264.7 macrophages with cell viability of ≥ 80% are considered safe [21]. In terms of NO inhibition, below 50% = weak activity [43], 50%–69% = good activity [43, 44], and above 70% = strong activity [44]. Extracts of *B. pinnatum* had the strongest NO inhibition activity, which was not due to toxicity with cell viability above 90%. Extracts of *K. gunniae* were also noncytotoxic at the highest concentration tested with good NO inhibition activity. However, the high NO inhibition shown by *M. undata* and *M. frangula* extracts was coupled with low cell viability, indicating toxicity rather than NO inhibition. Therefore, *K. gunniae* and *B. pinnatum* extracts, which had strong or good activity across all the antioxidant assays, the anti‐15‐LOX assay and the NO inhibition assay, and were noncytotoxic, were selected for the COX‐2 and cytokine regulation assays.

#### 4.2.2. Anti‐Inflammatory Assay Through Inhibition of COX‐2 Enzyme

Cyclooxygenase enzymes (COX‐1 and COX‐2), also known as prostaglandin synthases, catalyse the production of prostaglandins from arachidonic acid. Prostaglandins are one of the mediators of chronic inflammation [45]. In this study, COX‐2 inhibition was investigated to determine whether the plant extracts are able to inhibit the activities of the COX enzyme.

All the extracts had very good anti‐inflammatory activity against COX‐2 as they all significantly inhibited the production of this enzyme. However, extracts of *K. gunniae* were more active than extracts of *B. pinnatum* as the level of COX‐2 production was lower in cells treated with *K. gunniae* extracts, compared with cells treated with *B. pinnatum* extracts. This indicates that although *K. gunniae* and *B. pinnatum* are from the same family, extracts of *K. gunniae* and *B. pinnatum* most likely have different active plant constituents/compounds. Overall, *K. gunniae* extracts had strong inhibitory activity against COX‐2 and 15‐LOX, as well as strong antioxidant activity. *B. pinnatum* extracts had strong inhibitory activity against COX‐2 and NO. This indicates that the active compounds in *K. gunniae* extracts, and those in *B. pinnatum* extracts may exert their anti‐inflammatory activity through slightly different anti‐inflammatory pathways.

The COX‐2 pathway can be activated through either the arachidonic acid or the NO pathway. Through the arachidonic acid pathway, phospholipase A2 is stimulated by factors like leukotrienes/nitrogen species/ROS/excess prostaglandins/proinflammatory cytokines to cleave arachidonic acid from membrane phospholipids for metabolism, leading to the activation of the arachidonic acid inflammatory pathways of which COX‐2 is one [27]. Through the NO pathway, inducible iNOS is expressed after stimulation of cells with inflammatory mediators like LPS [42]. The iNOS then synthesises NO, and NO in turn modulates the activity of COX‐2 [21, 42].

Thus, it seems that the active constituents in *K. gunniae* extracts exert their COX‐2 inhibition mainly through the arachidonic acid pathway, whereas the active constituents in *B. pinnatum* extracts exert their COX‐2 inhibition activity mainly through the NO pathway. This then implies that for maximum efficacy, *K. gunniae* extracts will be more useful in treating chronic inflammation that is initiated mainly through the arachidonic acid pathway, whereas *B. pinnatum* extracts will be more useful in treating chronic inflammation that is initiated mainly through the NO pathway. Similar to the findings in this study that *B. pinnatum* extracts inhibited the activity of COX‐2, Zurfluh et al. [46] reported that the bufadienolide‐enriched fraction and bufadienolides from the leaf press juice of *B. pinnatum* inhibited COX‐2 expression. Fractionation of *K. gunniae* extracts as well as isolation of compounds and testing their antioxidant and anti‐inflammatory activities could help to identify the type of active plant secondary metabolites present, as well as understand whether the active constituents are acting in synergy.

#### 4.2.3. Effects of Extracts on Proinflammatory Cytokines, TNF‐α and IL‐1β

The presence of pathogens in the animal body can lead to upregulation of inflammatory reactions. The upregulation of inflammatory reactions then leads to the production of proinflammatory cytokines, and these in turn promote further upregulation of inflammatory reactions. This then leads to further production of inflammatory cytokines, leading to a stage known as a cytokine storm [47, 48]. TNF‐α is one of the proinflammatory cytokines, and uncontrolled production of TNF‐α is unhealthy [49]. In this study, extracts of *B. pinnatum* were able to significantly inhibit the production of TNF‐α, whereas extracts of *K. gunniae* had no significant effect on the inhibition of TNF‐α (Figure 4). As observed with the COX‐2 inhibition assay, the TNF‐α inhibition assay further shows that the extracts of *B. pinnatum* and *K. gunniae* act through different inflammatory pathways.

TNF‐α is one of the inflammatory mediators that can stimulate the expression of iNOS, which in turn synthesises NO that can possibly modulate the activity of COX‐2 [21, 42]. Therefore, following this pathway, any anti‐inflammatory agent that will be able to inhibit the production of TNF‐α will automatically inhibit the production of NO and COX‐2. The results of the anti‐inflammatory activity of *B. pinnatum* extracts strongly support this hypothesis. The results indicate that, as *B. pinnatum* extracts were able to inhibit the production of TNF‐α, they are consequently able to inhibit the production of NO and COX‐2 as observed in this study. This supports the supposition that the anti‐inflammatory activity of *B. pinnatum* occurs through a cytokine‐activated NO pathway.

Inhibition of cytokine IL‐1β, another proinflammatory cytokine [50], could be one of the ways that anti‐inflammatory agents exert their anti‐inflammatory activity on cells with pathological inflammation. Although the extracts in this study slightly decreased the level of IL‐1β production, the effect was statistically nonsignificant, indicating that the mechanism of anti‐inflammatory activity of extracts of *B. pinnatum* and *K. gunniae* is not through inhibition of IL‐1β.

#### 4.2.4. Effects of Extracts on Anti‐Inflammatory Cytokine IL‐10

The uncontrolled production of inflammatory cytokines aids the development of pathogenic inflammatory diseases [48]. A common feature of cytokines is that an activated cytokine can affect the production and activity of other cytokines in a synergistic, additive or antagonistic way [51]. If only proinflammatory cytokines are secreted and they function together in a synergistic or additive manner, inflammation will increase. A balance is needed in terms of the secretion of proinflammatory and anti‐inflammatory cytokines to maintain good health. A balance resulting in controlled inflammation can be achieved in secreted cytokines when there is an antagonistic effect of anti‐inflammatory cytokines towards proinflammatory cytokines [52].

The anti‐inflammatory cytokine IL‐10 controls inflammation through an immunosuppressive effect, which includes inhibition of macrophage activation, as well as having an antagonistic effect to proinflammatory cytokines by attenuating their expression [53]. An anti‐inflammatory agent that is able to increase the production of cytokine IL‐10 will help in establishing antagonistic effects towards proinflammatory cytokines, thereby discontinuing ongoing pathological inflammation. In this study, we determined the effect of the plant extracts on cytokine IL‐10. Extracts of *B. pinnatum* had very good positive activity on the production of the anti‐inflammatory cytokine IL‐10. The acetone extract of *B. pinnatum* significantly increased the production of IL‐10, and the ethanol extract of *B. pinnatum* maintained the production of IL‐10 at almost the same level as LPS‐activated cells. This indicates that the acetone and ethanol extracts of *B. pinnatum* most likely contain different plant active constituents and thus further supports the same observation seen with the effects of these extracts in the antioxidant assays and the 15‐LOX assay. These results further strongly affirm that extracts of *B. pinnatum* exert their anti‐inflammatory activity mainly through the cytokine‐activated NO pathway. Through this pathway, extracts of *B. pinnatum,* while inhibiting the production of the proinflammatory cytokine TNF‐α, at the same time increase the production of the anti‐inflammatory cytokine IL‐10 and these activities consequently trigger the inhibition of the production of NO and the proinflammatory enzyme COX‐2. However, for *B. pinnatum* ethanol extracts, the moderately increased production of IL‐10, the potent antioxidant activity and the potent 15‐LOX activity further indicate that the plant constituents of *B. pinnatum* ethanol extracts in particular act through an additional anti‐inflammatory pathway, which is the arachidonic acid pathway.

For *K. gunniae* extracts, the observation that they decreased the production of IL‐10 (although not to the low level of normal control cells) further shows that their anti‐inflammatory activity is neither through inhibition of proinflammatory cytokines nor promotion of anti‐inflammatory cytokines. The effect of *K. gunniae* extracts on IL‐10, their strong antioxidant activity and strong 15‐LOX activity further confirms that their anti‐inflammatory efficacy is mainly through the arachidonic acid pathway. However, although *K. gunniae* extracts do not increase the production of the anti‐inflammatory cytokine and do not inhibit the production of the proinflammatory cytokines TNF‐α and IL‐1β, their moderate inhibition of NO shows that they have slight activity through the NO pathway. It is possible that *K. gunniae* extracts may have some positive activities towards other proinflammatory and anti‐inflammatory cytokines not yet tested, and this is recommended for future studies.

### 4.3. Active *K. gunniae* and *B. pinnatum* Extracts: Potential for Managing Infectious Bovine Mastitis

In the present study, extracts of *K. gunniae* and *B. pinnatum* had promising antioxidant and anti‐inflammatory activities. In our previous research [16], the same extracts of these plants had excellent antibacterial activity against *Staphylococcus aureus* strains isolated from clinical cases of mastitis in dairy cows. Infectious mastitis is characterised by visible inflammation with redness, swelling and heat on the udder, with associated pain, which is indicative of inflammation resulting from the arachidonic acid pathway [54]. Inflammation that occurs through the NO pathway contributes to pain and vasodilation [55, 56]. Both the arachidonic acid and NO pathways are involved in infectious mastitis. In this study, the activity of these plant extracts indicates that they can inhibit infectious mastitis inflammation through both the arachidonic acid and NO pathways. Their combined antioxidant and anti‐inflammatory activity in addition to their activity against mastitis bacterial pathogens highlight them as strong candidates for the development of plant‐based preparations useful in treating or managing infectious bovine mastitis.

## 5. Conclusions

In this study, it was established that *Kalanchoe gunniae* and *Bryophyllum pinnatum* extracts have significant antioxidant and anti‐inflammatory activities. The extracts of the two plants appear to be effective via different anti‐inflammatory mechanisms, with *K*. *gunniae* exerting activity mainly through the arachidonic acid pathway and to some extent via the NO pathway. On the other hand, *B. pinnatum* acted mainly through the NO pathway and slightly through the arachidonic acid pathway.

Together with their known antibacterial efficacy against pathogens implicated in causing mastitis, the antioxidant and anti‐inflammatory activity of *K. gunniae* and *B. pinnatum* extracts supports continued research on these plants for their use as complementary medications against infectious bovine mastitis. Further work is needed to identify the active compounds responsible for the biological activities, and to ascertain whether the compounds in the extracts have activity as single compounds or if they work in synergy.

**Nomenclature**
Abs: absorbance
ABTS, 2: 2′‐azino‐bis (3‐ethylbenzothiazoline‐6‐sulfonic acid)
ANOVA: Analysis of variance
ATCC: American Type Culture Collection
*B. pinnatum*
: *Bryophyllum pinnatum*
CO_2_
: Carbon dioxide
COX: Cyclooxygenase
DMSO: Dimethyl sulfoxide
DMEM: Dulbecco’s modified Eagle’s medium
DPPH: 1,1‐Diphenyl‐2‐picrylhydrazyl
FOX reagent: (Preparation of xylenol orange and ferrous sulphate)
H.G.W.J.: Herold Georg Wilhelm Johannes
H_2_O_2_
: Hydrogen peroxide
IC_50_
: Concentration inhibiting 50% of the sample
IL‐1β: Interleukin‐1 beta
IL‐10: Interleukin‐10
iNOS: Inducible nitric oxide synthase
*K*. *gunniae*
: *Kalanchoe gunniae*
5‐LOX: 5‐Lipoxygenase
15‐LOX: 15‐Lipoxygenase
LPS: Lipopolysaccharide
*M. frangula*
: *Maurocenia frangula*
*M. undata*
: *Maytenus undata*
NA: Not applicable
L‐NAME: N‐nitro‐L‐arginine methyl ester
NaNO_2_
: Sodium nitrite
NO: Nitric oxide
NOS: Nitric oxide synthase
NF‐kB: Nuclear factor kappa B
OH: Hydroxyl
OOH, ROO: Peroxyl
ONOO‐: Peroxynitrite
PBS: Phosphate‐buffered saline
PLA_2_
: Phospholipase
PGE_2_
: Prostaglandin endoperoxide synthase 2
ROS: Reactive oxygen species
SCC: Somatic cell count
SANBI: South African National Biodiversity Institute
O_2_
: Superoxide
MTT: Methyl tetrazolium bromide
TNF‐α: Tumour necrosis factor

## Author Contributions

Dr E. C. Ogbuadike (ECO): conceptualisation of the research, conducted experiments, analysed results, initial drafting and writing of the manuscript.

Dr S. M. Nkadimeng (SMN): conducting of experiments, analysing results and initial drafting of manuscript.

Dr E. T. Khunoana (ETK): assisted with conducting experiments and the provision of some research materials (ELISA kits).

Dr C. C. Igwe (CCI): conceptualisation of the research, processing of one of the plant materials, proofreading of the initial draft.

Prof D. N. Qekwana (DNQ): research supervision, formatting and editing of manuscript.

Prof I. M. Petzer (IMP): research supervision, final proofreading and editing of manuscript.

Prof L. J. McGaw: research supervision, obtaining funding and editing of manuscript.

## Funding

Research funding was provided by the University of Pretoria (Translational Medicine Research Theme, Faculty of Veterinary Sciences) and the National Research Foundation (NRF, Grant number SRUG22051812086).

## Disclosure

The final manuscript was read and approved by all authors.

## Ethics Statement

The study was approved by the Research Ethics Committee of the Faculty of Veterinary Science, University of Pretoria (protocol certificate number REC124‐19).

## Conflicts of Interest

The authors declare no conflicts of interest.

## Data Availability

The data that support the findings of this study are available from the corresponding author upon reasonable request.

## References

[1] Banga C. , Neser F. , and Garrick D. , The Economic Value of Somatic Cell Count in South African Holstein and Jersey Cattle, South African Journal of Animal Science. (2014) 44, no. 2, 173–177, 10.4314/sajas.v44i2.10.

[2] Sharun K. , Dhama K. , Tiwari R. et al., Advances in Therapeutic and Managemental Approaches of Bovine Mastitis: A Comprehensive Review, Veterinary Quarterly. (2021) 41, no. 1, 107–136, 10.1080/01652176.2021.1882713. 33509059 PMC7906113

[3] Schukken Y. H. , Wilson D. J. , Welcome F. , Garrison-Tikofsky L. , and Gonzalez R. N. , Monitoring Udder Health and Milk Quality Using Somatic Cell Counts, Veterinary Research. (2003) 34, no. 5, 579–596, 10.1051/vetres:2003028, 2-s2.0-0242692449. 14556696

[4] Sargeant J. , Leslie K. , Shirley J. , Pulkrabek B. , and Lim G. , Sensitivity and Specificity of Somatic Cell Count and California Mastitis Test for Identifying Intramammary Infection in Early Lactation, Journal of Dairy Science. (2001) 84, no. 9, 2018–2024, 10.3168/jds.s0022-0302(01)74645-0, 2-s2.0-0035462982. 11573781

[5] Viguier C. , Arora S. , Gilmartin N. , Welbeck K. , and O’Kennedy R. , Mastitis Detection: Current Trends and Future Perspectives, Trends in Biotechnology. (2009) 27, no. 8, 486–493, 10.1016/j.tibtech.2009.05.004, 2-s2.0-67650932893. 19616330

[6] Harmon R. , Physiology of Mastitis and Factors Affecting Somatic Cell Counts, Journal of Dairy Science. (1994) 77, no. 7, 2103–2112, 10.3168/jds.s0022-0302(94)77153-8, 2-s2.0-0028472830. 7929968

[7] Swartz R. , Jooste P. J. , and Novello J. , Prevalence and Types of Bacteria Associated With Subclinical Mastitis in Bloemfontein Dairy Herds, Journal of the South African Veterinary Association. (1984) 55, no. 2, 61–64. 6492054

[8] Waage S. , Mørk T. , Røros A. , Aasland D. , Hunshamar A. , and Ødegaard S. , Bacteria Associated With Clinical Mastitis in Dairy Heifers, Journal of Dairy Science. (1999) 82, no. 4, 712–719, 10.3168/jds.s0022-0302(99)75288-4, 2-s2.0-0033114145. 10212457

[9] Iwalewa E. , McGaw L. , Naidoo V. , and Eloff J. , Inflammation: the Foundation of Diseases and Disorders. A Review of Phytomedicines of South African Origin Used to Treat Pain and Inflammatory Conditions, African Journal of Biotechnology. (2007) 6, no. 25.

[10] Colin D. A. and Monteil H. , Control of the Oxidative Burst of Human Neutrophils by Staphylococcal Leukotoxins, Infection and Immunity. (2003) 71, no. 7, 3724–3729, 10.1128/iai.71.7.3724-3729.2003, 2-s2.0-0037973528. 12819053 PMC161991

[11] Jalil S. , Mikhova B. , Taskova R. et al., In Vitro Anti-Inflammatory Effect of *Carthamus lanatus* L, Zeitschrift für Naturforschung C. (2003) 58, no. 11-12, 830–832, 10.1515/znc-2003-11-1215, 2-s2.0-0346966854. 14713160

[12] Sakat S. , Juvekar A. R. , and Gambhire M. N. , In Vitro Antioxidant and Anti-Inflammatory Activity of Methanol Extract of *Oxalis corniculata* Linn, International Journal of Pharmacy and Pharmaceutical Sciences. (2010) 2, no. 1, 146–155.

[13] Latha S. , Grace X. F. , Shanthi S. , Chamundeeswari D. , Seethalakshmi S. , and Reddy C. U. M. , In Vitro Antioxidant and Anti-Inflammatory Activity of Methanol Extract of *Stereospermum colais* (Buch.-Ham. ex. Dillw), Sri Ramachandra Journal of Medicine. (2011) 4, no. 1, 11–14.

[14] Adibhatla R. M. and Hatcher J. , Phospholipase A2, Reactive Oxygen Species, and Lipid Peroxidation in Cerebral Ischemia, Free Radical Biology and Medicine. (2006) 40, no. 3, 376–387, 10.1016/j.freeradbiomed.2005.08.044, 2-s2.0-31344432053. 16443152

[15] Paterson H. M. , Murphy T. J. , Purcell E. J. et al., Injury Primes the Innate Immune System for Enhanced Toll-Like Receptor Reactivity, Journal of Immunology. (2003) 171, no. 3, 1473–1483, 10.4049/jimmunol.171.3.1473, 2-s2.0-0041344362. 12874240

[16] Ogbuadike E. C. , Nkadimeng S. M. , Igwe C. C. et al., An in Vitro Study on the Potential of Selected South African Plant Extracts to Prevent and Treat Bovine Mastitis, South African Journal of Botany. (2023) 154, 98–107, 10.1016/j.sajb.2023.01.028.

[17] Sserunkuma P. , McGaw L. , Nsahlai I. , and Van Staden J. , Selected Southern African Medicinal Plants With Low Cytotoxicity and Good Activity Against Bovine Mastitis Pathogens, South African Journal of Botany. (2017) 111, 242–247, 10.1016/j.sajb.2017.03.032, 2-s2.0-85016418612.

[18] Eloff J. , Which Extractant Should be Used for the Screening and Isolation of Antimicrobial Components From Plants?, Journal of Ethnopharmacology. (1998) 60, no. 1, 1–8, 10.1016/s0378-8741(97)00123-2, 2-s2.0-0031885559. 9533426

[19] Gupta A. , Naraniwal M. , and Kothari V. , Modern Extraction Methods for Preparation of Bioactive Plant Extracts, International Journal of Applied and Natural Sciences. (2012) 1, no. 1, 8–26.

[20] Re R. , Pellegrini N. , Proteggente A. , Pannala A. , Yang M. , and Rice-Evans C. , Antioxidant Activity Applying an Improved ABTS Radical Cation Decolorization Assay, Free Radical Biology and Medicine. (1999) 26, no. 9-10, 1231–1237, 10.1016/s0891-5849(98)00315-3, 2-s2.0-0032982508. 10381194

[21] Nkadimeng S. M. , Nabatanzi A. , Steinmann C. M. , and Eloff J. N. , Phytochemical, Cytotoxicity, Antioxidant and Anti-Inflammatory Effects of *Psilocybe natalensis* Magic Mushroom, Plants. (2020) 9, no. 9, 10.3390/plants9091127. PMC7570254 32878164

[22] Bakasatae N. , Kunworarath N. , Yupanqui C. T. , Voravuthikunchai S. P. , and Joycharat N. , Bioactive Components, Antioxidant, and Anti-Inflammatory Activities of the Wood of *Albizia myriophylla* , Revista Brasileira de Farmacognosia. (2018) 28, no. 4, 444–450, 10.1016/j.bjp.2018.05.010, 2-s2.0-85048945329.

[23] Omisore N. , Adewunmi C. , Iwalewa E. et al., Antitrichomonal and Antioxidant Activities of *Dorstenia barteri* and *Dorstenia convexa* , Brazilian Journal of Medical and Biological Research. (2005) 38, no. 7, 1087–1094, 10.1590/s0100-879x2005000700012, 2-s2.0-23144455104. 16007280

[24] Phongpaichit S. , Nikom J. , Rungjindamai N. et al., Biological Activities of Extracts From Endophytic Fungi Isolated from *Garcinia* Plants, FEMS Immunology and Medical Microbiology. (2007) 51, no. 3, 517–525, 10.1111/j.1574-695x.2007.00331.x, 2-s2.0-35948941587. 17888010

[25] Brand-Williams W. , Cuvelier M. E. , and Berset C. , Use of a Free Radical Method to Evaluate Antioxidant Activity, Food Science and Technology. (1995) 28, no. 1, 25–30, 10.1016/s0023-6438(95)80008-5, 2-s2.0-58149364663.

[26] Pinto del Carmen M. , Tejeda A. , Duque A. L. , and Macías P. , Determination of Lipoxygenase Activity in Plant Extracts Using a Modified Ferrous Oxidation−Xylenol Orange Assay, Journal of Agricultural and Food Chemistry. (2007) 55, 5956–5959. 17602650 10.1021/jf070537x

[27] Ondua M. , Njoya E. M. , Abdalla M. A. , and McGaw L. J. , Anti-Inflammatory and Antioxidant Properties of Leaf Extracts of Eleven South African Medicinal Plants Used Traditionally to Treat Inflammation, Journal of Ethnopharmacology. (2019) 234, 27–35, 10.1016/j.jep.2018.12.030, 2-s2.0-85060465157. 30572091

[28] Adebayo S. A. , Dzoyem J. P. , Shai L. J. , and Eloff J. N. , The Anti-Inflammatory and Antioxidant Activity of 25 Plant Species Used Traditionally to Treat Pain in Southern African, BMC Complementary and Alternative Medicine. (2015) 15, no. 1, 10.1186/s12906-015-0669-5, 2-s2.0-84938974315. PMC4443658 26014115

[29] Mosmann T. , Rapid Colorimetric Assay for Cellular Growth and Survival: Application to Proliferation and Cytotoxicity Assays, Journal of Immunological Methods. (1983) 65, no. 1-2, 55–63, 10.1016/0022-1759(83)90303-4, 2-s2.0-0021061819. 6606682

[30] Noreen Y. , Ringbom T. , Perera P. , Danielson H. , and Bohlin L. , Development of a Radiochemical cyclooxygenase-1 and-2 in Vitro Assay for Identification of Natural Products as Inhibitors of Prostaglandin Biosynthesis, Journal of Natural Products. (1998) 61, no. 1, 2–7, 10.1021/np970343j, 2-s2.0-0031911266. 9461646

[31] du Toit K. , Elgorashi E. E. , Malan S. F. et al., Anti-Inflammatory Activity and QSAR Studies of Compounds Isolated From Hyacinthaceae Species and *Tachiadenus longiflorus* Griseb.(Gentianaceae), Bioorganic and Medicinal Chemistry. (2005) 13, no. 7, 2561–2568, 10.1016/j.bmc.2005.01.036, 2-s2.0-14844329083. 15755657

[32] Ahmed A. S. , McGaw L. J. , Elgorashi E. E. , Naidoo V. , and Eloff J. N. , Polarity of Extracts and Fractions of Four Combretum (Combretaceae) Species Used to Treat Infections and Gastrointestinal Disorders in Southern African Traditional Medicine Has a Major Effect on Different Relevant in Vitro Activities, Journal of Ethnopharmacology. (2014) 154, no. 2, 339–350, 10.1016/j.jep.2014.03.030, 2-s2.0-84901418230. 24681040

[33] Nkadimeng S. M. , Steinmann C. M. , and Eloff J. N. , Anti-Inflammatory Effects of Four Psilocybin-Containing Magic Mushroom Water Extracts in Vitro on 15-lipoxygenase Activity and on Lipopolysaccharide-Induced Cyclooxygenase-2 and Inflammatory Cytokines in Human U937 Macrophage Cells, Journal of Inflammation Research. (2021) 14, 3729–3738, 10.2147/jir.s317182. 34385833 PMC8352634

[34] Bhatti M. , Kamboj A. , Saluja A. K. , and Jain U. K. , In Vitro Evaluation and Comparison of Antioxidant Activities of Various Extracts of Leaves and Stems of *Kalanchoe pinnatum* , International Journal of Green Pharmacy. (2012) 6, no. 4, 10.4103/0973-8258.108255, 2-s2.0-84875956468.

[35] Abubakar A. R. and Haque M. , Preparation of Medicinal Plants: Basic Extraction and Fractionation Procedures for Experimental Purposes, Journal of Pharmacy and BioAllied Sciences. (2020) 12, no. 1, 1–10, 10.4103/jpbs.jpbs_175_19. 32801594 PMC7398001

[36] Wakeel A. , Jan S. A. , Ullah I. , Shinwari Z. K. , and Xu M. , Solvent Polarity Mediates Phytochemical Yield and Antioxidant Capacity of Isatis tinctoria, PeerJ. (2019) 7, 10.7717/peerj.7857, 2-s2.0-85074174154. PMC6790100 31616599

[37] Ahmed A. S. , McGaw L. J. , and Eloff J. N. , Evaluation of Pharmacological Activities, Cytotoxicity and Phenolic Composition of Four Maytenus Species Used in Southern African Traditional Medicine to Treat Intestinal Infections and Diarrhoeal Diseases, BMC Complementary and Alternative Medicine. (2013) 13, no. 1, 1–15, 10.1186/1472-6882-13-100, 2-s2.0-84877259049. 23663902 PMC3726504

[38] Smith G. F. , Figueiredo E. , Loureiro J. , and Crouch N. R. , *Kalanchoe × gunniae* Gideon F. Sm & Figueiredo (Crassulaceae), a New South African Nothospecies Derived From *Kalanchoe paniculata* Harv.× *Kalanchoe sexangularis* NE Br, Bradleya. (2019) 2019, no. 37, 141–150.

[39] Hu C. and Ma S. , Recent Development of Lipoxygenase Inhibitors as Anti-Inflammatory Agents, Medchemcomm. (2018) 9, no. 2, 212–225, 10.1039/c7md00390k, 2-s2.0-85042684539. 30108915 PMC6083793

[40] Dzoyem J. P. , Nkuete A. H. , Ngameni B. , and Eloff J. N. , Anti-Inflammatory and Anticholinesterase Activity of Six Flavonoids Isolated From *Polygonum* and *Dorstenia* Species, Archives of Pharmacal Research. (2017) 40, no. 10, 1129–1134, 10.1007/s12272-015-0612-9, 2-s2.0-84930536002. 26048035

[41] Ojewole J. A. , Antinociceptive, Anti-Inflammatory and Antidiabetic Effects of *Bryophyllum pinnatum* (Crassulaceae) Leaf Aqueous Extract, Journal of Ethnopharmacology. (2005) 99, no. 1, 13–19, 10.1016/j.jep.2005.01.025, 2-s2.0-17644421061. 15848014

[42] Salvemini D. , Misko T. P. , Masferrer J. L. , Seibert K. , Currie M. G. , and Needleman P. , Nitric Oxide Activates Cyclooxygenase Enzymes, Proceedings of the National Academy of Sciences. (1993) 90, no. 15, 7240–7244, 10.1073/pnas.90.15.7240, 2-s2.0-0027249753. PMC47112 7688473

[43] Ana B. , Abdul M. A. , Radzali M. , and Noorjahan B. M. , Anti-Oxidant and Anti-Inflammatory Activities of Leaves of *Barringtonia racemosa* , Journal of Medicinal Plants Research. (2007) 1, no. 5, 095–102.

[44] Yang E.-J. , Yim E.-Y. , Song G. , Kim G.-O. , and Hyun C.-G. , Inhibition of Nitric Oxide Production in Lipopolysaccharide-Activated RAW 264.7 Macrophages by Jeju Plant Extracts, Interdisciplinary Toxicology. (2009) 2, no. 4, 245–249, 10.2478/v10102-009-0022-2, 2-s2.0-77954549348. 21217861 PMC2984114

[45] Sales K. J. and Jabbour H. N. , Cyclooxygenase Enzymes and Prostaglandins in Pathology of the Endometrium, Reproduction. (2003) 126, no. 5, 559–567, 10.1530/rep.0.1260559, 2-s2.0-0346752324. 14611628 PMC2695735

[46] Zurfluh L. , Santos S. , Ruppen A. et al., Bryophyllum pinnatum Modulation of Signaling Pathways Relevant for Preterm Labor in Human Myometrial Cells, Biomedicine and Pharmacotherapy. (2025) 184, 10.1016/j.biopha.2025.117919. 39983434

[47] Pasare C. and Medzhitov R. , Toll-Like Receptors: Linking Innate and Adaptive Immunity, Mechanisms of Lymphocyte Activation and Immune Regulation. (2005) 6, 11–18. 10.1007/0-387-24180-9_2 15932016

[48] Tang X.-D. , Ji T.-T. , Dong J.-R. et al., Pathogenesis and Treatment of Cytokine Storm Induced by Infectious Diseases, International Journal of Molecular Sciences. (2021) 22, no. 23, 10.3390/ijms222313009. PMC8658039 34884813

[49] Dinarello C. A. , The Proinflammatory Cytokines interleukin-l and Tumor Necrosis Factor and Treatment of the Septic Shock Syndrome, Journal of Infectious Diseases. (1991) 163, no. 6, 1177–1184, 10.1093/infdis/163.6.1177, 2-s2.0-0026006260. 2037782

[50] Dinarello C. A. , Proinflammatory Cytokines, Chest. (2000) 118, no. 2, 503–508, 10.1378/chest.118.2.503, 2-s2.0-0033874108. 10936147

[51] Turrin N. P. and Plata-Salamán C. R. , Cytokine–Cytokine Interactions and the Brain, Brain Research Bulletin. (2000) 51, no. 1, 3–9, 10.1016/s0361-9230(99)00203-8, 2-s2.0-0033966866. 10654575

[52] Chaudhry H. , Zhou J. , Zhong Y. et al., Role of Cytokines as a Double-Edged Sword in Sepsis, In Vivo. (2013) 27, no. 6, 669–684. 24292568 PMC4378830

[53] Steen E. H. , Wang X. , Balaji S. , Butte M. J. , Bollyky P. L. , and Keswani S. G. , The Role of the Anti-Inflammatory Cytokine interleukin-10 in Tissue Fibrosis, Advances in Wound Care. (2020) 9, no. 4, 184–198, 10.1089/wound.2019.1032. 32117582 PMC7047112

[54] Ricciotti E. and FitzGerald G. A. , Prostaglandins and Inflammation, Arteriosclerosis, Thrombosis, and Vascular Biology. (2011) 31, no. 5, 986–1000, 10.1161/atvbaha.110.207449, 2-s2.0-79955586225. 21508345 PMC3081099

[55] Giles T. D. , Sander G. E. , Nossaman B. D. , and Kadowitz P. J. , Impaired Vasodilation in the Pathogenesis of Hypertension: Focus on Nitric Oxide, Endothelial‐Derived Hyperpolarizing Factors, and Prostaglandins, Journal of Clinical Hypertension. (2012) 14, no. 4, 198–205, 10.1111/j.1751-7176.2012.00606.x, 2-s2.0-84859332979. 22458740 PMC8108814

[56] Gomes F. I. F. , Cunha F. Q. , and Cunha T. M. , Peripheral Nitric Oxide Signaling Directly Blocks Inflammatory Pain, Biochemical Pharmacology. (2020) 176, 10.1016/j.bcp.2020.113862. 32081790

